# International benchmarking of tertiary trauma centers: productivity and throughput approach

**DOI:** 10.1186/1752-2897-5-10

**Published:** 2011-08-03

**Authors:** Antti Peltokorpi, Lauri Handolin, Matthias Frank, Paulus Torkki, Gerrit Matthes, Axel Ekkernkamp, Eero Hirvensalo

**Affiliations:** 1 Institute of Healthcare Engineering, Management and Architecture, BIT Research Centre, Aalto University, Otaniementie 17, 00076 Aalto, Finland; 2 Töölö Hospital, Helsinki and Uusimaa Hospital District, Topeliuksenkatu 5, Helsinki, Finland; 3 Unfallkrankenhaus Berlin, Warener Straße 7, 12683 Berlin, Germany

## Abstract

**Background:**

Care process in tertiary trauma centers consists of a chain of care phases in different departments from the emergency department (ED) to post-operative rehabilitation. The historical evolution of healthcare systems and organizations has led to variations in trauma patient processes in different countries. The present study is aimed at revealing differences in the throughput and productivity of trauma patient processes between German (UKB) and Finnish (HUS) tertiary trauma centers. Problems related to the comparison of different healthcare systems were also identified. The share of patients discharged was used as a control measure.

**Results:**

The biggest differences between the hospitals were found in the use of resources in the ED and in post-operative care. Despite problems in defining comparable patients and resources, ED productivity was significantly higher in UKB. Post-operative care was, on average, 41% shorter in HUS. However, the share of patients discharged was significantly higher in UKB (96.5% vs. 68.9%). Differences were also found in the pre-operative length of stay of patients with proximal femoral fractures (UKB: 0.97 days, HUS: 1.57 days). The productivity of the operating unit was quite similar in the hospitals. In terms of ED mortality, no statistically significant differences were found.

**Conclusions:**

The results of the present study showed significant differences in the use of resources and throughput times in trauma patient processes between Finnish and German hospitals. However, due to system-level differences between German and Finnish healthcare, the results cannot be directly transformed into development proposals for the organizations. On the other hand, in spite of certain differences regarding the healthcare systems, the demographic data of the trauma patients and medical procedures are comparable. Based on the present study, the ED process of severe trauma, pre-operative care, and operating unit processes were the most comparable parts of trauma care between the hospitals. The study also showed that the international benchmarking approach could be used to reveal bottlenecks in system-level policies and practices.

## Background

Producing excellent patient care outcomes and maintaining high productivity at the same time are especially challenging in trauma hospitals. Tertiary trauma centers usually have one of the most complex case mixes among hospitals: Instead of process-based care protocols, a remarkable number of patients have to be cared as a project due to the number and severity of their injuries. In light of work by Schmenner [1], from a service operations management point of view, project-based care means that the variation in resource use is large between patients and between trauma centers.

A reliable comparison of trauma patient care is possible only between hospitals with a similar focus and patient mix. Since tertiary trauma centers are typically responsible for taking care of the most severe injuries in the center's regional catchment area as well as from even longer distances (e.g., surrounding rural areas), finding similar hospitals inside the same region is typically not possible. Therefore, analyzing the performance with benchmarking has to be understood in a wider context, such as comparing trauma centers in metropolitan areas and internationally [2]. International benchmarking could also reveal bottlenecks in healthcare system-level policies that should be discussed in a wider forum [3]. Such benchmarking could provide evidence of socially optimal structures in the provision and financing of healthcare [4].

In the area of healthcare, international benchmarking was first applied during the 1980s at the policy and system levels by the OECD and WHO [3]. Since then, the approach has been used more and more in comparing hospitals [2,4,5] and care processes [6]. In recent studies, international benchmarking has been extended to also cover trauma centers. Gabbe et al. [7] compared outcomes following major trauma in an inclusive trauma system (Victoria, Australia) and a setting where rationalization of trauma services is absent (England and Wales). Schuetz et al. [8] benchmarked trauma care performance in a tertiary hospital in Queensland and in European trauma centers. In trauma benchmarking studies, mortality has been one of the most frequently used outcome measures, [7-9] whereas throughput time before arrival at the hospital, in the emergency department (ED), and of in-patient care have been used as process measures [8,10,11]. Also, productivity measures, such as the number of patients seen by a physician and the nurse-physician ratio, have been widely used [11-14].

Based on previous research, most remarkable differences between hospitals have been seen in the length of stays and the use of intensive care units (ICU) of comparable trauma patients. However, clear reasons for these differences are seldom identified. In the present study, the productivity and throughput of the trauma patient process were benchmarked between Finnish and German tertiary trauma centers. The primary focus is on identifying practices that explain differences in productivity and throughput. Care outcome was considered a secondary measure. Special attention was paid in discussions with the hospital managers to the observed differences in order to ascertain the underlying reasons.

## Methods

### Study settings

The present study is aimed at revealing features in organizational structures and management practices that enable higher productivity and throughput of trauma patient care. The essential question is to analyze how many resources were used to care for comparable trauma patients in different hospitals and the main reasons behind the differences. The outcome of care was analyzed only to verify that the current managerial practices were not destructive to care quality and patient safety.

Since the aim of the study is to conduct a deep performance analysis of several phases in trauma patient care, only two tertiary trauma centers, Unfallkrankenhaus Berlin (UKB) in Germany and Töölö Acute Trauma Hospital (HUS) in Finland, were benchmarked. The hospitals were selected based on their representativeness as a tertiary trauma center in a metropolitan area and their project cooperation with Aalto University. The trauma patient was defined as someone with a trauma injury who needs surgical care, either operative or non-operative, within three weeks. Trauma patient care was considered in four phases: emergency department care, pre-operative care, operating unit care, and post-operative care. In the ICU the analysis was restricted to comparing the number of beds and personnel resources per trauma patient. In the present study, the benchmarking method focused on evaluating performance in the two hospitals and revealing the factors behind the perceived performance.

### Case environments

Both benchmarked trauma centers have a high academic research and teaching status.

UKB is one of the largest tertiary trauma centers in Germany. The hospital consists of a coordinated structure of 20 specialized departments and institutes. UKB's primary catchment area is about 260,000 inhabitants. In addition, it serves patients in the whole region of Berlin (3.3 million inhabitants) with five other trauma hospitals nationally. Each year, more than 49,000 emergency patients are treated at UKB, of whom more than 12,000 are admitted for in-patient care. The hospital provides jobs for 1,260 employees, and it has 538 beds and 13 operating rooms (ORs). It also has emergency service vehicles and a helicopter based on the top of the hospital building.

Töölö Hospital (HUS) is the largest trauma center in Finland. This hospital is responsible for caring for patients with severe trauma in the southern part of Finland and certain specific patient groups nationally. The primary catchment area of the hospital is about 600,000 inhabitants, and the tertiary area, about 1.5 million inhabitants. The hospital consists of five disciplines: orthopedics and traumatology, hand surgery, neurosurgery, plastic and reconstructive surgery, and acute oral and maxillofacial surgery. About 19,000 patients are treated annually in the ED, of whom 7,000 are admitted to the hospital. The hospital has 192 ward beds and 14 operating rooms (ORs).

### Study variables and data gathering

Trauma patient care consists of a chain of care phases provided by several units inside the hospital: the ED, ICUs, ORs, and pre- and post-operative ward units. When comparing the total performance of the trauma patient process, it is important to analyze the whole chain in the hospital and how the overall performance consists of partial productivities and outcomes in different care phases [15].

**Figure 1:**
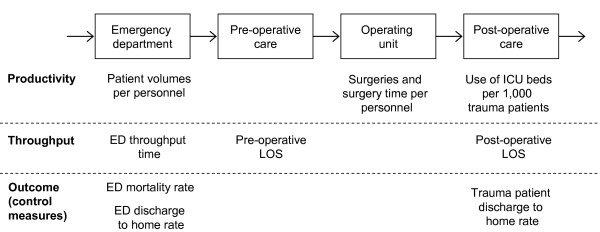
**The investigated trauma patient process and used performance measures**.

According to previous studies [16], the most relevant benchmarking indicators are related to patient group-based measures, such as length of stay, and productivity measures, which take into account the ratio between the resources used and the output produced [4]. The study variables of the present study focus on the productivity and throughput of the trauma patient process (Figure 1 and Table 1). Productivity was defined as the ratio between the number of patients receiving care and the personnel resources used. In the ED, patients were also categorized as admitted and non-admitted patients and as surgical and non-surgical patients due to an assumption that surgical or admitted patients consume more resources than discharged patients. In the operating unit, different surgeries were standardized based on their average surgery time. In terms of resources, partial productivities were calculated for work performed by surgeons, anesthesiologists, and nurses.

**Table 1:** Study variables used in the benchmarking study

*Category*	*Measures for all trauma patients*	*Measures for patients with proximal femoral fractures*
Basic information	ED visits per year (n) ISS ≥15/22 patients per year (n) Trauma team activations per year (n) ED visits admitted to hospital (n/%) Acute surgeries (n/%) Acute trauma surgeries (n/%)	Surgeries per year (n) Mean patient age (years)
Productivity measures	ED volumes per physician/nurse/total personnel in ED (n) Acute surgeries per physician/nurse/total personnel in ED (n) Trauma surgeries per surgeon/anesthesiologist/nurse in operating units (n) Trauma surgery time per surgeon/anesthesiologist/nurses in operating units (n)	
Throughput measures	Average pre-operative length of stay (LOS) per urgency category (hours) Share of trauma patients operated within first/second day (%) Average post-operative LOS (days) Average total LOS (days)	Average pre-operative LOS (hours) Average post-operative LOS (days) Average surgery time (min)
Outcome measures	ED mortality rate (excluding death-on-arrival patients) (%) Share of non-admitted ED patients (%) Share of trauma patients discharged from ward (%)	Share of patients discharged (%)

Throughput was defined as a patient's length of stay during the care phase. Throughput times were calculated separately for the ED process, pre-operative stay in the hospital, and post-operative care. Due to the different urgencies and recovery processes of different trauma patients, throughput times were calculated separately for patients with proximal femoral fracture, who accounted for one of the most common shared patient groups in the hospitals.

The study variables were divided into four categories: basic information, outcome measures, productivity measures, and throughput measures. Basic information measures were used to reveal variations in case mixes and hospital profiles. The underlying assumption was that patients with high ISS or trauma team activation need more resources than walking patients. The ED mortality rate and the share of patients discharged were used as the outcome measures of trauma care.

The study material consisted of data from interviews, process documents, observations, and information systems. Hospital personnel from the ED, ward units, and operating units were interviewed about management responsibilities, personnel resources, care processes and practices, responsibilities for care decisions, space and facilities, patient transfers, and planning methods. Interviews and discussions with hospital management were also used for mapping system-level features, including ownership and funding, organization of care, and catchment areas.

Documents were gathered from all patient groups whose care process was systematically mapped. Observations included visits by HUS doctors to UKB and researchers' observations in the EDs, wards, and ORs in both hospitals. The aim of the observations was to verify that the mapped processes worked as planned and to make remarks about the resources and process practices that were not mentioned in the interviews.

The process data of patients served during a 12-month period was gathered retrospectively from the hospital databases. The UKB data included patients in 2006 and the HUS data included patients in 2008. The different time periods were due to the project cooperation schedule. The data included patient diagnoses, procedures, urgencies, unit and OR identification, surgeon identification, and the next time stamps of the OR process and unit transfers: patient arrives in the ED, patient discharged from the ED, patient transferred to the OR, surgery starts, surgery ends, patient discharged from the OR, patient discharged from the hospital.

## Results

Basic information about the case mix and patient volumes is presented in Table 2. The total acute surgery volumes are nearly the same. Patient volumes in the ED are significantly higher in UKB, meaning that UKB also provides services for non-surgical patients. Based on the number of trauma team activations, however, the EDs' patient severity profile is quite similar.

**Table 2:** Basic information about the case hospitals

*Basic information measures*	*UKB*	*HUS*
ED visits per year	49,000	19,360

ISS ≥15 patients per year	-	450 (2.32%)

ISS ≥22 patients per year	-	250 (1.29%)

Trauma team activations per year (n/% of all visits)	900 (1.84%)	400 (2.07%)

ED admissions to the hospital (n/%)	12,400 (25%)	6,960 (36%)

Acute surgeries (n/%)	6,520 (13%)	6,800 (35%)

Acute O&T surgeries (n/%)	2,280 (4.7%)	3,550 (18%)

The productivity, throughput, and outcome measures of the hospitals are presented in Table 3. Nurse and secretary productivity in the ED, measured both as visits and decisions for surgery per personnel, were higher in UKB (Table 3). Physician productivity, though, was higher in HUS when the output was measured as decisions for surgery. The factor behind the contradictory findings was staffing practices: UKB tended to have the same number of physicians and nurses per shift in the ED, whereas there were four times more nurses than physicians in HUS. Nurse productivity in operating units was a little higher in UKB when the output was defined as surgery hours. In orthopedics and traumatology (O&T), however, surgeon productivity was higher in HUS when the surgery length-related definition was used for the output. Pre-operative LOSs were a little shorter in UKB. Total LOS, however, in HUS was only half of that in UKB.

**Table 3:** Performance measures of the whole process

*Measure*	*UKB*	*HUS*
ED visits per physician workday	11.2	8.84

ED visits per nurse and secretary workday	8.95	1.77

Decisions for surgery per ED physician workday	1.49	3.58

Decisions for surgery per ED nurse and secretary workday	1.19	0.72

Surgeries per year per anesthesiologist	862	695

Surgeries per year per nurse in operating unit	154	90.4

Surgery hours per year per anesthesiologist	786	1120

Surgery hours per year per nurse in operating unit	151	136

O&T surgeries per specialist	177	174

O&T surgery hours per specialist	241	314

Pre-operative LOS, emergency patients [hours]	6:13	7:15

Pre-operative LOS, other urgent patients [days]	2.86	3.73

Share of trauma patients operated on within the first day	52.7%	35.0%

Share of trauma patients operated on within two days	73.9%	54.6%

Total LOS, all surgical patients [days]	7.1	4.2

Total LOS, O&T surgical patients [days]	8.6	4.3

ED mortality rate (excluding death-on-arrival patients)	0.082%	0.052%

Share of non-admitted ED patients (%)	75%	64%

Share of trauma patients discharged from ward	96.5%	68.9%

Mortality in the ED was a little higher in UKB. About two thirds of the ED patients were discharged in both hospitals. One of the biggest differences was found in the discharge rate after surgery: Only 3.5% of O&T surgical patients at UKB were transferred to another hospital, whereas more than 30% at HUS were transferred. UKB seemed to rehabilitate patients more than HUS, and this was also seen in patients' significantly longer stays (LOSs) in UKB.

More specific measures for patients with proximal femoral fractures are presented in Table 4. Pre-operative LOS was significantly shorter in UKB, whereas post-operative LOS and surgery time were shorter in HUS. Only 15% of the patients were transferred to another hospital from UKB; and only 80% patients from HUS.

**Table 4:** Performance in the care process of patients with proximal femoral fractures

*Measure*	*UKB*	*HUS*
Surgeries per year	241	657

Mean patient age [years]	63.6	79.5

Share of patients discharged	85.8%	20.6%

Pre-operative LOS [days]	0.97	1.57

Post-operative LOS [days]	11.86	5.34

Surgery time [h:mm]	1:23	1:14

The hospitals' system-level features as well as process and management practices are illustrated in Table 5. The largest differences relate to funding and competition. Those differences are also reflected in management practices and processes. For example, UKB admits all patients who need care to the ED, whereas HUS applies tight patient selection based on the severity of the illness and specialty needed. UKB also aims at maximizing patient condition in the hospital's own care processes, but at HUS, the patient is rehabilitated to a certain degree and then transferred to another hospital.

**Table 5:** Main remarks about the features in the system level, processes, and management practices in the study hospitals

*Level of analysis*	*Subject*	*UKB*	*HUS*
System-level features	Ownership & funding	Worker's foundation owned academic hospital, primarily for occupational injuries, but not exclusive 60% of patients occupationally insured, others with private or social insurance	Public-funded university hospital 100% of patient care funded by municipalities' income taxes
	Organization of care	All trauma patients (referred and non-referred) are taken care of	Primary trauma care is usually not taken care of; mostly, referred patients are seen
	Catchment area	Primary: 260,000 Secondary: 3.3 M (competing for customers with 5 tertiary trauma centers); calculated average, 550,000 Tertiary: 7.7 M (competing for customers with 9 tertiary trauma centers); calculated average, 770,000	Primary: 600,000 Secondary: 600,000 Tertiary: 1.5 M
Process and management features of trauma patients	Acute patient volumes	ED visits: 49,000 per year ⇒ ED admissions to hospital: 12,400 (25%) ⇒ Acute surgeries: 6,520 (for 4,660 patients) ⇒ Acute O&T surgeries: 2,280 (for 1,780 patients)	ED visits: 19,400 per year ⇒ ED admissions to hospital: 6,960 (36%) ⇒ Acute surgeries: 6,800 (for 5,140 patients) ⇒ Acute O&T surgeries: 3,550 (for 2,990 patients)
	Emergency department	All patients admitted to the ED. Rapid response and patient categorization highlighted in the reception Lean approach applied, especially in trauma team activations IT system supports rapid response and shift to the next phase of care	Non-severe primary traumas are directed to other hospitals Regional and national responsibility over care after catastrophes highlighted by the management Focus on maintaining capacity and readiness to receive multi-traumas and multiple patient scenarios in any circumstances
	Pre-operative care	Patient transferred directly to an operating room or a ward unit Ward care conducted primarily in sub-specialty-focused ward units. High flexibility, however, between wards to accommodate patients from other sub-specialties	Most patients transferred to a dedicated ward unit for pre-operative trauma patients. Emergencies transferred directly to an operating room "Green line" is used a lot to discharge less severe trauma patients from the ED and to schedule a surgery in a defined operating room session within several days
	Surgical care	Large multi-specialty operating unit. In addition, a couple of operating rooms for day surgeries Anesthesia induction conducted in a separate room in every surgery One anesthesiologist is responsible for one operating room	Dedicated operating units for O&T, neurosurgery, plastic and reconstructive surgery, and day surgery Anesthesia induction conducted in a separate room in a small part of surgeries One anesthesiologist is responsible for one to three operating rooms
	Post-operative care	Conducted in the same ward as pre-operative care. Integrated rehabilitation care; almost all patients are discharged to home	Immediate post-operative care conducted in wards dedicated to certain injuries of different body parts Rehabilitation conducted mainly in communal hospitals

The main differences in trauma care performance between UKB and HUS can be summarized in the following two findings:

1) Total length of stay is significantly shorter in HUS

2) ED productivity is higher in UKB

Based on the discussion and evaluation of the results with the hospital managers, several underlying reasons for the differences were identified. First, HUS transferred many patients to regional hospitals that preside over the rehabilitation process. This policy is a result of limited bed capacity in trauma hospitals, Finnish municipalities' aim to use their own lower-cost hospital capacity for non-specialized care, and a trend towards rehabilitating patients near their homes. Second, in Germany, insurance policy defines the minimum length of stay for most cases. This was proposed to explain the proportionally longer LOSs in UKB, especially for minor traumas. However, at present, the goal is to adopt shorter LOSs like those seen in Finland. ED productivity was higher in UKB primarily due to leaner nurse staffing. Discussions with hospital managers demonstrated that UKB has a more suitable ED layout. Managers also argued that differences in patient profiles could partially explain the higher resource availability in HUS's ED.

## Discussion

The aim of the study is to identify differences in the performance of trauma hospitals and to reveal the variables in the system environment, strategic decisions, and operational practices behind those differences. The results show reasons for the differences at all three levels.

At the system level, many differences are related to funding and its effect, e.g., on hospital stays. The study results show that both non-integrated special and primary care and conditions of insurance could have a limiting effect on the efficiency of organizations and care processes. In addition, differences in nurse staffing in the ED can partially be explained by education and labor market issues at the system level. Based on recent statistics, there are 50% more nurses per capita in Finland compared to Germany [17]. That statistic was also reflected in the study results. When benchmarking more hospitals from several countries, it can be assumed that more variables in the system environment that affect overall performance will be found. Differences at the system level should be presented to the politicians and managers responsible for addressing such issues.

Although the phases of the trauma care process were quite similar in the hospitals, remarkable differences were found in the use of resources during each phase. The study also revealed that the trauma care process has universal primary objectives, but the sub-objectives differ between units. For example, the EDs in both hospitals are focused very intensively on a short care process and rapid transfer to the next care phase. However, their resource composition was quite different, focusing on physicians in Germany and on nurses in Finland. As a result, resource use per patient was different. Similarly, the surgical units in both hospitals focused strongly on high resource utilization and dedicated personnel per OR session. That led to quite similar productivity results in the surgical units. This might also indicate that the productivity approach is more thoroughly applied globally in a surgical unit environment than in other departments in hospitals.

The high variation in post-operative care between the hospitals was noteworthy in this study. At a more general level, the result raises the question as to whether international differences in resource use and productivity are higher in less acute and less protocol-based care phases such as rehabilitation than in emergency and precise care. The results of previous benchmarking studies support this conclusion [4]. Rehabilitation is more strongly connected with system-level and society features, such as the occupational health system and relatives' support, than more medically oriented pre-operative examinations and surgical operations. Those features can also affect diversified resource use and processes in rehabilitation between countries. Statistics also show that there are more hospital beds per capita in Germany compared to Finland [17]. However, the difference is not as major as in the duration of post-operative care between the trauma centers, reflecting that in Finland the role of regional hospitals is emphasized in patient rehabilitation.

With respect to post-operative care, this study was limited to comparing the use of resources, LOS, and rehabilitation results only in tertiary trauma centers. Therefore, in the future, research should include all the units providing post-operative care in the comparisons. There is also a need for studies analyzing and comparing qualitative rehabilitation results more thoroughly.

The power of the international comparison utilized in this study is that it can reveal performance differences to a higher degree than differences between organizations in the same region. This increases the possibility that the difference is not invalidated by practitioners due to, e.g., accuracy issues, but is responded to seriously. Although there are always some data compatibility problems, international benchmarking makes identifying practices that enable remarkable improvements instead of marginal changes possible.

## Conclusions

The study showed that international benchmarking can be a potential source for new practices and improvements in healthcare. As Booth et al. [2] argue, benchmarking within healthcare should be seen as indicating areas that may be looked at in further detail. The benchmarking of trauma centers is not the end of the story, merely the beginning.

## Competing interests

The authors declare that they have no competing interests.

## Authors' contributions

AP coordinated the writing process and was responsible for the data analysis and literature review. LH participated in the design of the study, coordinated the data gathering at HUS, and formulated the features of the hospitals. MF coordinated the data gathering at UKB and participated in the writing process. PT analyzed data and participated in the writing process. GM participated in the design of the study and was responsible for the research tasks conducted at UKB. AE participated in the design of the study. EH participated in the design of the study and supervised the research tasks conducted at HUS. All authors read and approved the final manuscript.
